# Active Kras Expression in Gastric Isthmal Progenitor Cells Induces Foveolar Hyperplasia but Not Metaplasia

**DOI:** 10.1016/j.jcmgh.2018.09.007

**Published:** 2018-09-18

**Authors:** Eunyoung Choi, Anna L. Means, Robert J. Coffey, James R. Goldenring

**Affiliations:** 1 Nashville VA Medical Center, Vanderbilt University School of Medicine, Nashville, Tennessee; 2 Section of Surgical Sciences, Vanderbilt University School of Medicine, Nashville, Tennessee; 3 Epithelial Biology Center, Vanderbilt University School of Medicine, Nashville, Tennessee; 4 Department of Medicine, Vanderbilt University School of Medicine, Nashville, Tennessee; 5 Department of Cell and Developmental Biology, Vanderbilt University School of Medicine, Nashville, Tennessee; 6 Vanderbilt-Ingram Cancer Center, Vanderbilt University School of Medicine, Nashville, Tennessee

**Keywords:** Clu, clusterin, EGFR, epidermal growth factor receptor, GIF, gastric intrinsic factor, GSII, Griffonia simplicifolia lectin II, pERK1/2, phospho-extracellular signal–regulated kinase 1/2, SPEM, spasmolytic polypeptide expressing metaplasia, TGFα, transforming growth factor α, UEAI, Ulex europaeus

Two types of metaplasia are associated with the development of intestinal-type cancers in the human stomach: intestinal metaplasia and spasmolytic polypeptide (Trefoil Factor 2 [TFF2])-expressing metaplasia (SPEM). Previous investigations have noted activation of RAS protein in up to 40% of human gastric cancer patients.^1^ Our recent results using the *Mist1*^*CreERT*^*;Kras* ^*LSL-G12D*^ mice, expressing constitutively active Kras^G12D^ in Mist1-expressing cells (Mist-Kras mice), showed that induced expression of active *Kras* in chief cells can rapidly lead to SPEM, intestinal metaplasia, and invasive lesions.^2^ Thus, these studies suggested that active Ras in chief cells could drive the full spectrum of metaplastic transitions. Nevertheless, a conflicting paradigm of metaplasia origin has been suggested, proposing that rare Mist1-expressing isthmal progenitor cells serve as the origin of metaplasia.^3^ Despite other investigations that have supported the origin of SPEM from chief cells,^4^, ^5^ we have sought to evaluate directly the effects of expression of constitutively activated Kras (G12D) specifically in isthmal progenitor cells using the *Lrig1*^*CreERT2*^ mouse allele.^6^

We previously reported that the *Lrig1*^*CreERT2*^ mouse allele showed expression in isthmal progenitor cells without detectable expression in chief cells.^6^ The *Lrig1*^*CreERT2*^;*LSL-Kras*^*G12D*^ (Lrig1-Kras) mouse facilitated induction of active Kras expression only in isthmal progenitor cells in the gastric corpus. Two months after tamoxifen injection, we observed hyperplastic glands with parietal cell loss, an expanded foveolar cell region, and mature chief cells at the gland bases, while the *Lrig1*^*CreERT2*^ (Lrig1) mouse stomach mucosa was normal after tamoxifen injection (Figure 1*A*, white arrows). The chief cells still contained darkly stained basophilic cytoplasm, indicating that chief cells were not affected by induction of active Kras expression in isthmal progenitor cells.

**Figure 1:**
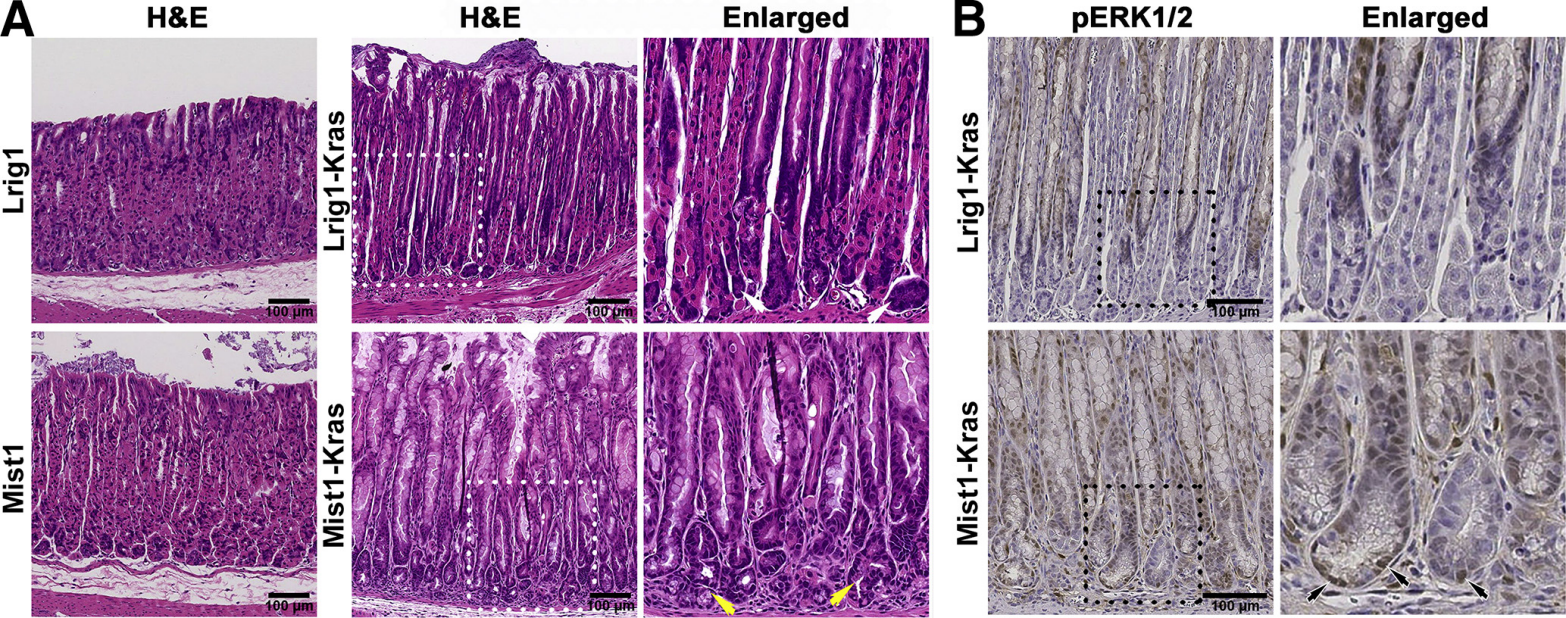
**Histologic staining of the stomachs of Lrig1-Kras or Mist1-Kras mice.** (*A*) Sections from Lrig1 or Lrig1-Kras mouse stomachs at 2 month after tamoxifen injection or from Mist1 or Mist1-Kras mouse stomachs at 1 month after tamoxifen injection were examined by H&E staining. *Dotted boxes* indicate enlarged regions. *Yellow arrows* indicate eosinophilic SPEM cells, which contain clear cytoplasm filled with mucous. *White arrows* indicate chief cells, which contain darkly stained basophilic cytoplasm. (*B*) Sections of the stomach corpus from Lrig1-Kras or Mist1-Kras mice were stained for pERK1/2 immunostaining. *Dotted boxes* indicate enlarged regions. *Black arrows* indicate pERK1/2-positive SPEM cells at the base of metaplastic glands.

In contrast, in Mist1-Kras mice, at just 1 month after tamoxifen treatment we observed evolution of eosinophilic SPEM cells, which contained a clear cytoplasm filled with mucus, at the bases of glands in the mouse corpus (Figure 1*A*, yellow arrows). The active Kras expression in chief cells also was associated with the development of foveolar cell hyperplasia in the gastric glands toward the lumen. Gland fission was observed at the bases of the glands, which is consistent with clonal expansion of metaplastic glands (Figure 1*A*, yellow arrows).

To assess Kras expression in the corpus, we examined phospho-extracellular signal–regulated kinase 1/2 (pERK1/2) expression in Mist1-Kras and Lrig1-Kras mouse stomachs, as an indication of induction of active Kras expression in the mucosa (Figure 1*B*). The pERK1/2-positive cells were observed throughout metaplastic glands in the Mist1-Kras mouse stomach corpus and the expression of pERK1/2 was detected in SPEM cells at the base of the metaplastic glands (Figure 1*B*, black arrows). However, the pERK1/2 expression in the Lrig1-Kras mice was detected only in the proliferative cell zone and above, but not at the base of the glands where the chief cells were located, confirming that the constitutively active Kras expression was targeted to the isthmal progenitor cells in the Lrig1-Kras mouse stomachs.

To compare the pathologic mucosal changes between Mist1-Kras and Lrig1-Kras mouse stomachs, we performed co-staining using Ulex europaeus (UEAI) lectin, a surface mucous cell marker, along with the SPEM markers Griffonia simplicifolia lectin II (GSII) lectin and an antibody against gastric intrinsic factor (GIF) (Figure 2*A*), or with antibodies against the SPEM marker, CD44 variant 9 (CD44v9) and the cellular stress marker, Clusterin (Clu) (Figure 2*E*). UEAI labeled expanded surface cells both in the Mist1-Kras and Lrig1-Kras gastric mucosa. In the Mist1-Kras mouse stomachs, the cells at the base of the glands were co-positive for GSII and GIF along with CD44v9 and Clu, confirming the presence of GIF+/GSII+ and CD44v9+/Clu+ dual-positive SPEM cells (Figure 2*A* and *E*, white arrows). However, the cells at the bases of glands in the Lrig1-Kras mouse stomachs were positive only for GIF (GIF+/GSII-), confirming that they were mature chief cells and no SPEM cells were detected.

**Figure 2:**
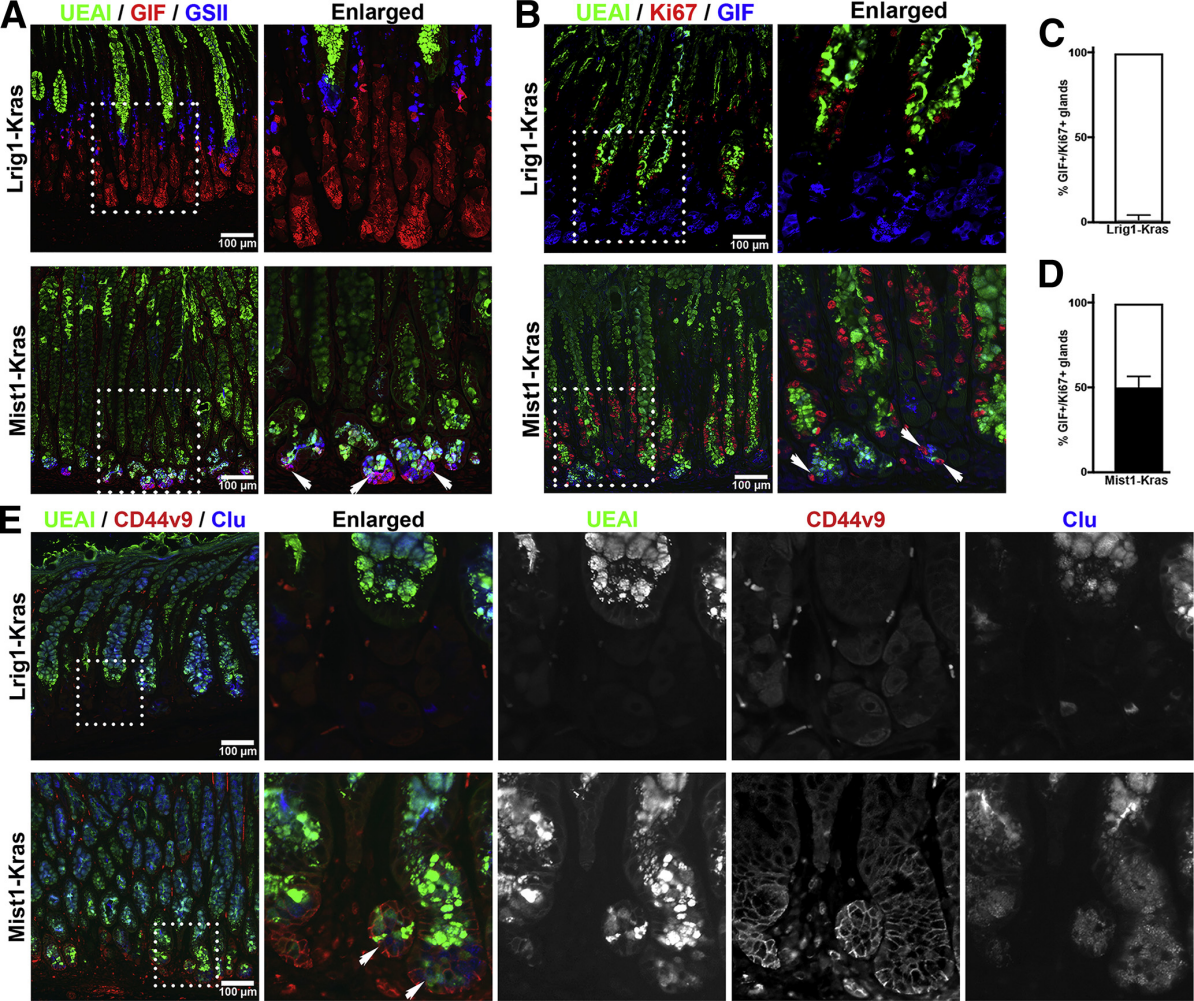
**Immunofluorescence of foveolar cell or SPEM markers in Lrig1-Kras or Mist1-Kras mouse stomachs.** (*A*) Sections of the stomach corpus from Lrig1-Kras or Mist1-Kras mice were co-stained for UEAI, GSII, and GIF. UEAI+ cells indicate foveolar cells, GSII+ cells are mucous neck cells, and GIF+ cells are mature chief cells. Dual GIF+/GSII+ cells indicate SPEM cells (*white arrows*). *Dotted boxes* indicate enlarged regions. (*B*) Sections of the corpus from Lrig1-Kras or Mist1-Kras mouse stomachs were immunostained with antibodies against Ki67, a proliferating cell marker, with UEAI and GIF. *White arrows* indicate proliferating SPEM cells at the base of metaplastic glands and *dotted boxes* indicate enlarged regions. Quantitation of GIF+/Ki67+ cells containing glands per 20× field in (*C*) Lrig1-Kras or (*D*) Mist1-Kras mouse stomach corpus. A total of 1.55% of glands contained GIF+/Ki67+ cells in Lrig1-Kras mouse corpus and 49.92% of glands contained GIF+/Ki67+ cells in Mist1-Kras mouse corpus. *Error bars* indicate SD (N = 3). (*E*) Sections of the stomach corpus from Lrig1-Kras or Mist1-Kras mice were co-stained for UEAI, CD44v9, and Clu. Cells with only UEAI staining indicate foveolar cells and dual CD44v9+/Clu+ cells indicate SPEM cells (*white arrows*). *Dotted boxes* indicate enlarged regions.

We also evaluated the presence of proliferating cells in the mucosa. In the Mist1-Kras mice, the Ki67-positive proliferative cells were broadly distributed from the neck region of glands to the base. The Ki67-positive cells in the neck region were co-positive for UEAI and Ki67 (UEAI+/Ki67+), while cells at the base of the glands were co-positive for GIF (GIF+/Ki67+), consistent with the presence of proliferative SPEM (Figure 2*B*, white arrows) in approximately 50% of total glands (Figure 2*D*) as well as proliferative foveolar cells. However, the position of the proliferative cell zone in the Lrig1-Kras stomach glands was restricted to the lower neck zone of hyperplastic glands and Ki67 co-localized with UEAI (UEAI+/Ki67+), but not with GIF (GIF-/Ki67+) (Figure 2*C*). Thus, cells present in the hyperplastic glands of Lrig1-Kras mouse corpus were proliferative foveolar cells, but not SPEM cells.

In this study, although the expression of active Kras in chief cells led to both foveolar hyperplasia and SPEM development, the lineages derived from active Kras induced in isthmal progenitor cells were only foveolar cells, but not SPEM cells. Isthmal Lrig1+ cells in the Lrig1-Cre^ERT2^ mouse can give rise to all lineages in the corpus mucosa,^6^ although the existence of Lrig1-negative isthmal progenitors cannot be ruled out presently. Parietal cell atrophy was observed both in Mist1-Kras and Lrig1-Kras mouse corpus. An increase of gastrin associated with acute parietal cell atrophy causes preferential expansion of foveolar cells.^7^ Previous studies of molecular mechanisms underlying the massive foveolar hyperplasia observed in a mouse model of Ménétrier’s disease (transforming growth factor α [TGFα]) showed that overexpression of TGFα and stimulation of the epidermal growth factor-receptor (EGFR) promoted isthmal progenitor cell differentiation preferentially into foveolar cells.^8^ Similar results also were observed in Ménétrier’s disease.^8^ Importantly, treatment of Ménétrier’s disease patients with antibodies against the EGFR led to amelioration of the foveolar hyperplasia, with decreases in phosphorylation of EGFR and ERK1/2, as well as return of gastric parietal cells.^9^ Thus, the Ménétrier’s disease scenario is consistent with selective activation of foveolar hyperplasia through stimulation of EGFR and activation of the Ras/ERK pathway. This paradigm of foveolar hyperplasia is similar to that shown here for Lrig1-Kras mice and recent findings in the eR1-Kras mice,^10^ which showed a predominance of foveolar hyperplasia in response to Kras activation in isthmal progenitor cells.

The present studies suggest that expression of active Kras in isthmal progenitors results in expansion of the surface mucous cell compartment, but not development of SPEM. The findings all point to the presence of homeostatic progenitor cell populations in the isthmus that are distinct from the ability of chief cells to transdifferentiate into SPEM after significant mucosal injury.

## Supplementary Material

### Animal Models

The care, maintenance, and treatment of mice used in this study followed protocols approved by the Institutional Animal Care and Use Committee of Vanderbilt University and each experimental group contained 3–4 mice. The generation of *Mist1-Kras*, *Mist1-CreERT2*^*Tg/+*^; *LSL-K-ras(G12D)*^*Tg/+*^, mice have been described previously.^1^, ^2^, ^3^ To induce active Kras in gastric stem/progenitor cells, *Lrig1-CreERT2/+* mice were bred with *LSL-K-ras(G12D)*^*Tg/+*^ mice. All the *Mist1-CreERT2*^*Tg/+*^, *Lrig1-CreERT2*^*Tg/+*^, or *LSL-K-ras(G12D)*^*Tg/+*^ mice were maintained on a C57BL6 background and both male and female mice were used for studies. For Mist1-Kras mouse studies, 5 mg of tamoxifen (Sigma, St. Louis, MO) in corn oil with 10% ethanol was administered to Mist1-Kras mice at 8 weeks of age by subcutaneous injection for 3 consecutive days, and they were killed 1 month after the tamoxifen injection (n = 3). For Lrig1-Kras mouse studies, 2 mg of tamoxifen in corn oil was administered to Lrig1-Kras mice at 8 weeks of age by intraperitoneal injection and they were killed 2 months after the tamoxifen injection (n = 3).

### Tissue Preparation and Antibody Staining

Tissue preparation and subsequent antibody staining of formalin-fixed, paraffin-embedded tissues were performed as previously described.^4^ Briefly, mouse stomachs from Mist1-Kras and Lrig1-Kras mice were fixed overnight in 4% paraformaldehyde solution (Affymetrix, Santa Clara, CA) and embedded in paraffin. For antibody staining, paraffin sections were deparaffinized in Histoclear solution (Electron Microscopy Sciences, Hatfield, PA) and rehydrated in a series of ethanol washes. Antigen retrieval was performed using citrate buffer, pH 6.0 (Sigma, St. Louis, MO), using a pressure cooker and blocked in Serum-Free Protein Block solution (Dako, Santa Clara, CA) at room temperature for 1.5 hours. Primary antibodies, goat anti-GIF (1:2000; a gift from Dr David Alpers), rabbit anti-Ki67 (1:1000; Cell Signaling, Danvers, MA), rabbit anti-pERK1/2 (1:1000; Cell Signaling, Danvers, MA), rat anti-CD44v9 (1:25,000; a gift from Professor H. Saya), and goat anti-clusterin (1:2000; Sigma) diluted in Antibody Diluent with Background Reducing Components (Dako) were incubated at 4°C overnight. Fluorescein isothiocyanate–conjugated lectin from UEAI (1:2000; Sigma), Alexa 647–conjugated GSII lectin (1:2000; Molecular Probes, Eugene, OR), and fluorescent secondary antibodies (1:500; Invitrogen, Carlsbad, CA) were incubated at room temperature for 1 hour. 4′,6-Diamidino-2-phenylindole was used to detect nuclei in immunofluorescence images. For immunohistochemistry, pERK1/2 antibody was detected with the Dako Envision+ System Horseradish Peroxidase 3,3′-diaminobenzidine tetra hydrochloride (Dako North America, Inc, Santa Clara, CA). All fluorescence images were acquired using a Zeiss Axio Imager M2 microscope (Oberkochen, Germany), equipped with a SPOT Explorer camera and using SPOT basic software (Sterling Heights, MI). For pERK1/2 immunohistochemistry, all stained stomach paraffin tissues were scanned using a Leica Versa automated slide scanner, Wetzlar, Germany at 20× magnification. Image overlay and preparation was performed in Adobe Photoshop (San Jose, CA). The means or SD of the means of quantitation data were generated using Prism 7 (GraphPad, La Jolla, CA).
